# Performance of IRI 2016 model in predicting total electron content (TEC) compared with GPS-TEC over East Africa during 2019–2021

**DOI:** 10.1038/s41598-024-59624-0

**Published:** 2024-05-01

**Authors:** Emmanuel D. Sulungu

**Affiliations:** Department of Physics, The University of Dodoma, Dodoma, Tanzania

**Keywords:** IRI model, GPS, Total electron content (TEC), Space physics, Physics

## Abstract

This study evaluated the applicability of IRI-2016 model in predicting GPS TEC using the monthly means of the five (5) quiet days for equinoxes and solstices months. GPS-derived TEC data were obtained from the IGS network of ground based dual frequency GPS receivers from three stations [(KYN3 0.53° S, 38.53° E; Geom. Lat. 3.91.63° S), (MBAR 0.60° S, 30.74° E; Geom. Lat. 2.76° S) and HOID 1.45° S, 31.34° E; Geom. Lat. 3.71° S]. All the three options for topside *Ne* of IRI-2016 model and ABT-2009 for bottomside thickness have been used to compute the IRI TEC. The results were compared with the GPS TEC measurements. Correlation Coefficients between the two sets of data, the Root-Mean Square Errors of the IRI-TEC from the GPS-TEC, and the percentage RMSE of the IRI-TEC from the GPS-TEC have been computed. In general, the IRI-2016 model underestimated GPS-TEC during the nighttime, whereas the model overestimated GPS-TEC values during the daytime. At most of the stations and during all seasons where data were available, correlation coefficient was above 0.9, which is quite strong. The variation of O/N2 ratio may potentially be the cause of the IRI TEC deviation from the GPS TEC. This variation arises from lower thermosphere plasma drift that moves upward.

## Introduction

The ionosphere is a part of the Earth’s atmosphere, which ranges from about 50 km–1000 km in altitude. There are a lot of free ions and electrons in it, which may affect how radio waves propagate. The equatorial and low-latitude ionosphere is very dynamic because of various phenomena, including the equatorial spread-F (ESF) irregularities, the equatorial electrojet (EEJ), and the equatorial ionization anomaly (EIA) or Appleton anomaly^1–3^. In the daytime, the EIA is the most distinct feature in the low-latitude ionosphere. It happens when the strong upward **E × B** drift of plasma at high altitudes is caused by the daylight eastward electric field near the geomagnetic equatorial ionosphere. Subsequently, the plasma descends and expands in both directions along geomagnetic field lines, forming a trough at the equator and two crests situated roughly 15 degrees apart from the magnetic equator. The dynamic nature of the low latitude ionosphere has significant impacts on radio signals used for navigation and communication. When a signal from a GPS satellite travel through the ionized layer in the ionosphere, it experiences refraction or bending, which changes its trajectory^3^.

An important quantity that represents the disturbance of the ionosphere of the Earth is the total electron content (TEC). TEC is described as the integral of the electron density along the ray path from the satellite to the receiver, with units of electrons per square meter, where 1 TEC unit (TECU) = 10^16^ electrons/m^2^. It can be used to estimate and improve the precision and dependability of impacted communication and navigation systems^4^. TEC changes as a function of geographic location, time of the day, day of the season, season of the year, and solar and geomagnetic activities^5^. The GPS has been used all around the world to study the variation of TEC in the ionosphere^6–9^. In an area where measured data are unavailable, ionospheric empirical models such as the International Reference Ionosphere (IRI) model play a significant role in the estimation of ionospheric TEC. IRI started in 1968/69 as a joint permanent scientific project of the Committee on Space Research (COSPAR) and the International Union of Radio Science (URSI)^3,10^. IRI model has been continually upgraded and this process has resulted in improved versions of IRI, and the IRI-2020 is the latest version^11^. However, this study will compare GPS TEC with IRI 2016 since the data used are from 2019, before the establishment of the IRI 2020 version.

Several studies have been done worldwide to compare the results from GPS TEC measurements and that from IRI model^4,10,12–18^. On the other hand, Amaechi et al.^12^ conducted a study on comparisons of the ionospheric anomalies over African equatorial/low-latitude region with IRI-2016 model predictions during the maximum phase of solar cycle 24. Tariku^19^ studied the mid latitude ionospheric TEC in comparison with IRI TEC model during high solar activity (2013–2015). Also, Okoh^10^ compared TEC predictions from the IRI model with TEC observations from the AFRL-SCINDA GPS receiver station at Nsukka, Nigeria. Nethertheless, comparisons between observed TEC and TEC from IRI 2016 model over the southern crest of the EIA, which lies inside the Eastern Africa region, are scarce. Therefore, this study aimed to evaluate the applicability of IRI-2016 model in predicting GPS TEC using data recorded at three stations within the region for the solar minimum 2019–2021.

## Data source and methods of analysis

### TEC from GPS receivers

**Figure 1:**
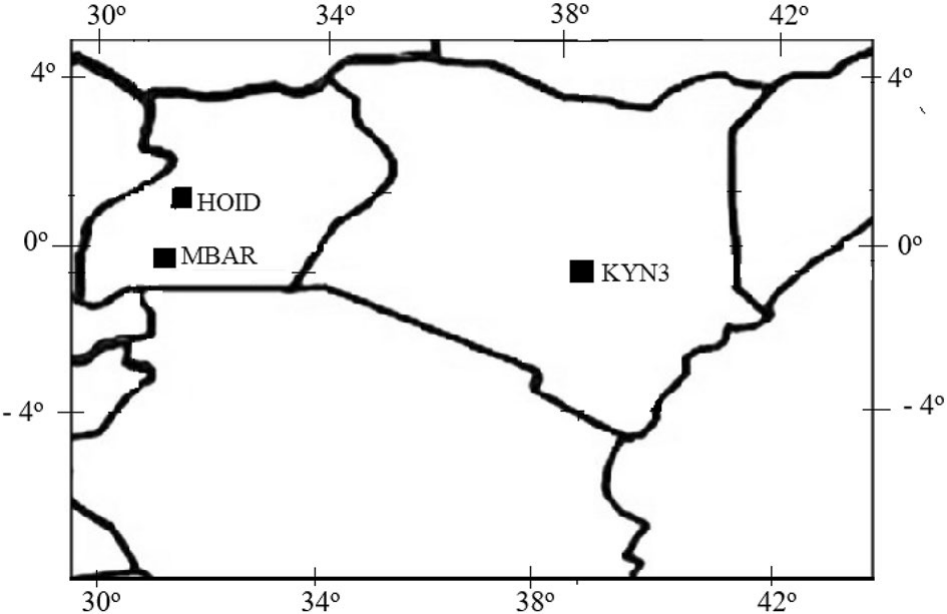
The map view showing the stations used in this study.

The GPS TEC data used in this study were obtained from three dual frequency GPS receivers within the East African region (Fig. 1) for the period of solar minimum 2019–2021. Table 1 shows the geographical and geomagnetic coordinates of these stations. The GPS data were obtained from the University NAVSTAR Consortium (UNAVCO) website (https://www.unavco.org/data/gps-gnss/data-access-methods/dai1/perm_sta.php). The GPS observable data were retrieved in standard Receiver Independent Exchange (RINEX) format and processed by the GPS-TEC processing software^20^. The total electron content measurements were obtained from dual-frequency receivers at L1 (1575.42 MHz) and L2 (1227.60 MHz) frequencies. The receivers measure slant TEC (sTEC) obtained from the difference between the pseudo-ranges (P1 and P2) and the difference between the phases (L1 and L2) of the two signals^21^ using (1) and (2).

1 $$sTEC_{L} = \left[ {\left( {\frac{{f_{2}^{2} }}{{f_{1}^{2} - f_{2}^{2} }}} \right)\frac{{2f_{1}^{2} }}{K}} \right]\left( {P_{2} - P_{1} } \right)$$

2 $$sTEC_{P} = \left[ {\left( {\frac{{f_{2}^{2} }}{{f_{1}^{2} - f_{2}^{2} }}} \right)\frac{{2f_{1}^{2} }}{K}} \right]\left( {L_{1} \lambda_{1} - L_{2} \lambda_{2} } \right)$$

where K = 80.62 m^3^s^−2^ is a constant that links plasma frequency to electron density, *f*_1_ and *f*_2_ are the high and low GPS frequencies, respectively, and λ_1_ and λ_2_ are the wavelengths that correspond to those frequencies.

**Table 1:** Stations coordinates.

Station	Code	Geographic	co-ordinates	Magnetic	Co-ordinates
		Latitude	Longitude	Latitude	Longitude
Garba Tula	KYN3	0.53° S	38.53° E	3.91° S	110.87° E
Mbarara	MBAR	0.60° S	30.74° E	2.76° S	103.16° E
Hoima	HOID	1.45° S	31.34° E	3.71° S	103.60° E

In order to compare the electron contents for pathways with various elevation angles, the STEC delivered at a sample rate of 30 s are transformed into equivalent vertical TEC (VTEC) using a mapping function that takes the curvature of the Earth into account as follows:

3 $$VTEC = \left[ {1 - \frac{{\cos \left( e \right)^{2} }}{{1 + {\raise0.7ex\hbox{$h$} \!\mathord{\left/ {\vphantom {h {R_{E} }}}\right.\kern-0pt} \!\lower0.7ex\hbox{${R_{E} }$}}}}} \right]^{\frac{1}{2}} \times STEC - \left( {b_{s} + b_{r} + b_{rx} } \right)$$

where *b*_*s*_ is the satellite differential delay (satellite bias), *b*_*r*_ is the receiver differential delay (receiver bias), *b*_*rx*_ is a receiver interchannel bias, *e* is an elevation angle of a satellite, *h* is ionospheric shell height, taken as 400 km in this study, and R_E_ is the Earth's mean radius. GPS-TEC software used to calculate VTEC reads raw data, processes cycle slips in phase data, reads satellite biases from International GNSS Service (IGS) code file, if not available, it calculates them, calculates receiver bias, and calculates the interchannel biases for different satellites in the receiver. To neglect unwanted errors due to the effect of multipath, a cut off elevation angle of 30° was used. Because many VTEC measurements are acquired at once from various satellites, a running average is used to produce a single curve for a day.

In this work, an hourly mean of VTEC data for the international geomagnetic quiet days only from equinoxes and solstices months has been taken because the IRI model predictions are best during the geomagnetic quiet days^12,13,22^. The mean of VTEC on five (5) international geomagnetic quiet days from each month taken into consideration has been used in order to obtain the monthly mean. The World Data Center for Geomagnetism, Kyoto, publishes the international geomagnetic quiet days at http://wdc.kugi.kyoto-u.ac.jp/cgi-bin/qddays-cgi.

### TEC from IRI 2016 model

The IRI model is one of the most widely used models proposed for international application by the Committee on Space Research (COSPAR) and the International Union of Radio Science (URSI), and it is primarily used to specify the ionospheric parameters. The IRI model is updated on regular basis by the scientific community, and the IRI-2016 model incorporates major improvement for electron density representation as well as other parameters. To get TEC from the IRI model, the precise location, date, and time were fed as inputs to the model. The IRI 2016 is obtained from the IRI website https://ccmc.gsfc.nasa.gov/modelweb/models/iri2016_vitmo.php. The IRI-2016 model is available in three different options for bottomside thicknesses (Bil-2000, Gul-1987, and ABT-2009) and three options for the topside electron density (*Ne*) (IRI-NeQuick, IRI-2001, and IRI-01-Corr). In this study, the ABT-2009 option for the bottom-side thickness parameter and the URSI option for the F2 peak density were utilized to produce the hourly values of the TEC from the IRI-2016 model utilizing all the three options of topside *Ne*. The use of ABT-2009 model was chosen because it predicts B_0_ more accurately than the Bil-2000 model by up to 32% and up to 40% over the Gul-1987 model. Additionally, it has up to 20% improvement for the B_1_ values^3,23^. Further, TEC data from IRI model employing URSI coefficients were chosen in this study because it performs well over the locations with scarce data measurements like oceans and the southern hemisphere region which mostly lies within African region^3^.

Correlation Coefficients (*r*) between the two data sets, the Root-Mean Square Errors (RMSE) of the IRI-TEC from the GPS-TEC, and the percentage RMSE (PRMSE) of the IRI-TEC from the GPS-TEC have been computed using (4)–(6).

4 $$r = \frac{{\sum\nolimits_{i} {\left( {GPS_{i} - \overline{GPS} } \right)\left( {IRI_{i} - \overline{IRI} } \right)} }}{{\sum\nolimits_{i} {\left( {GPS_{i} - \overline{GPS} } \right)^{2} \sum\nolimits_{i} {\left( {IRI_{i} - \overline{IRI} } \right)^{2} } } }}$$

5 $$RMSE = \sqrt {\frac{{\sum\nolimits_{i = 1}^{n} {\left( {GPS_{i} - IRI_{i} } \right)^{2} } }}{n}}$$

6 $$PRMSE = \frac{RMSE}{{\sqrt {\frac{{\sum\nolimits_{i = 1}^{n} {\left( {GPS_{i} } \right)^{2} } }}{n}} }} \times 100$$

where *GPS*_*i*_ are GPS-TEC data, $\overline{GPS}$ is their mean, *IRI*_*i*_ are IRI-TEC data, $\overline{IRI}$ is their mean, and *n* is the number of them, and the subscripts ‘*i*’ denote numerical positions in the data, having integral values from 1 to *n*.

## Results and discussion

### Comparison of GPS TEC with the TEC from IRI-2016 model

**Figure 2:**
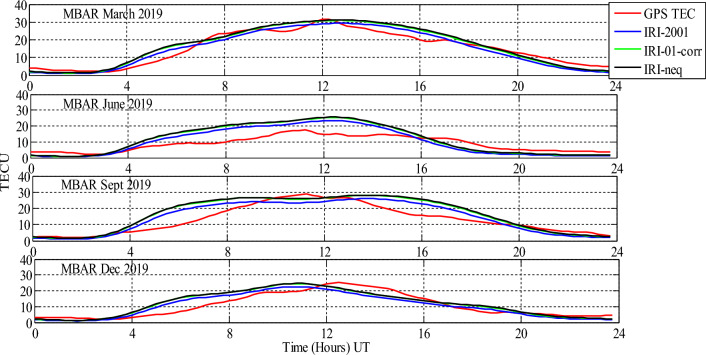
Comparison of GPS TEC and IRI-2016 model TEC from the monthly means of the 5 quietest days of the equinoxes and solstices months at MBAR for 2019.

**Figure 3:**
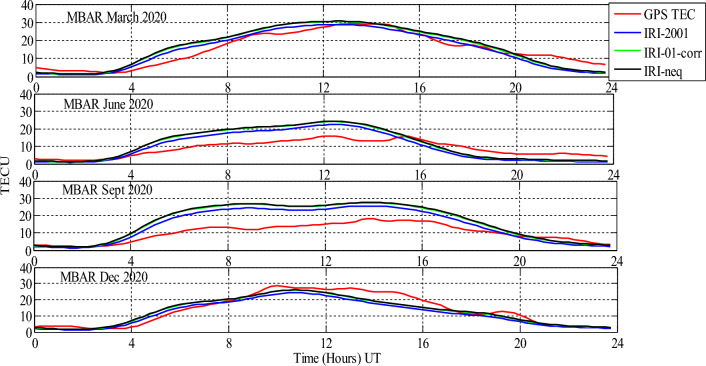
Comparison of GPS TEC and IRI-2016 model TEC from the monthly means of the 5 quietest days of the equinoxes and solstices months at MBAR for 2020.

**Figure 4:**
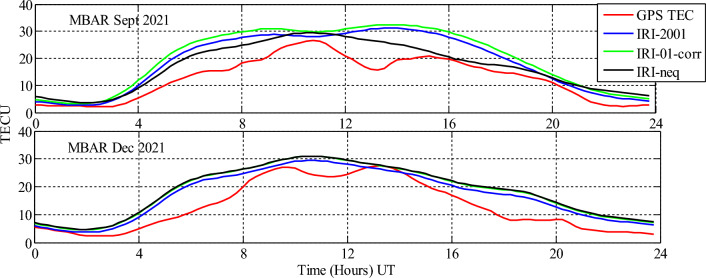
Comparison of GPS TEC and IRI-2016 model TEC from the monthly means of the 5 quietest days of the equinoxes and solstices months at MBAR for 2021.

**Figure 5:**
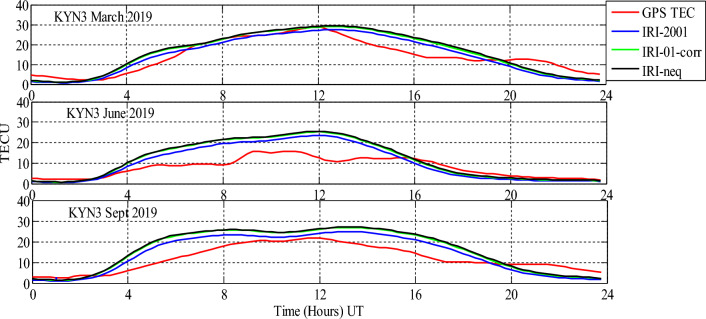
Comparison of GPS TEC and IRI-2016 model TEC from the monthly means of the 5 quietest days of the equinoxes and solstices months at Garba Tula (KYN3) for 2019.

**Figure 6:**
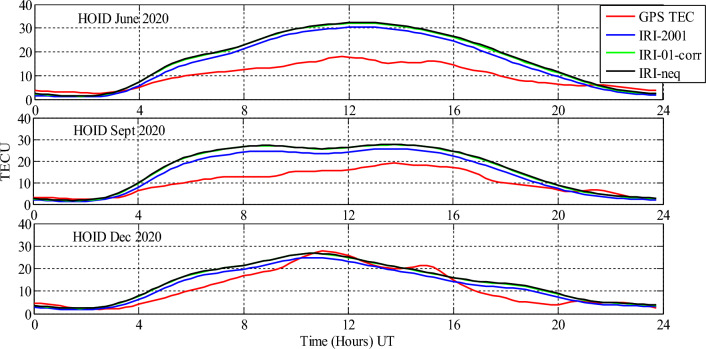
Comparison of GPS TEC and IRI-2016 model TEC from the monthly means of the 5 quietest days of the equinoxes and solstices months at HOID for 2020.

**Figure 7:**
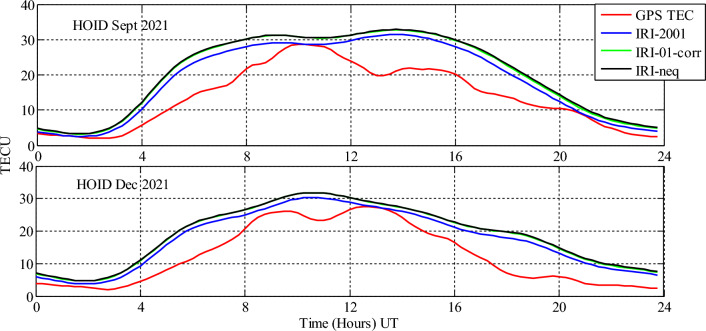
Comparison of GPS TEC and IRI-2016 model TEC from the monthly means of the 5 quietest days of the equinoxes and solstices months at HOID for 2021.

**Figure 8:**
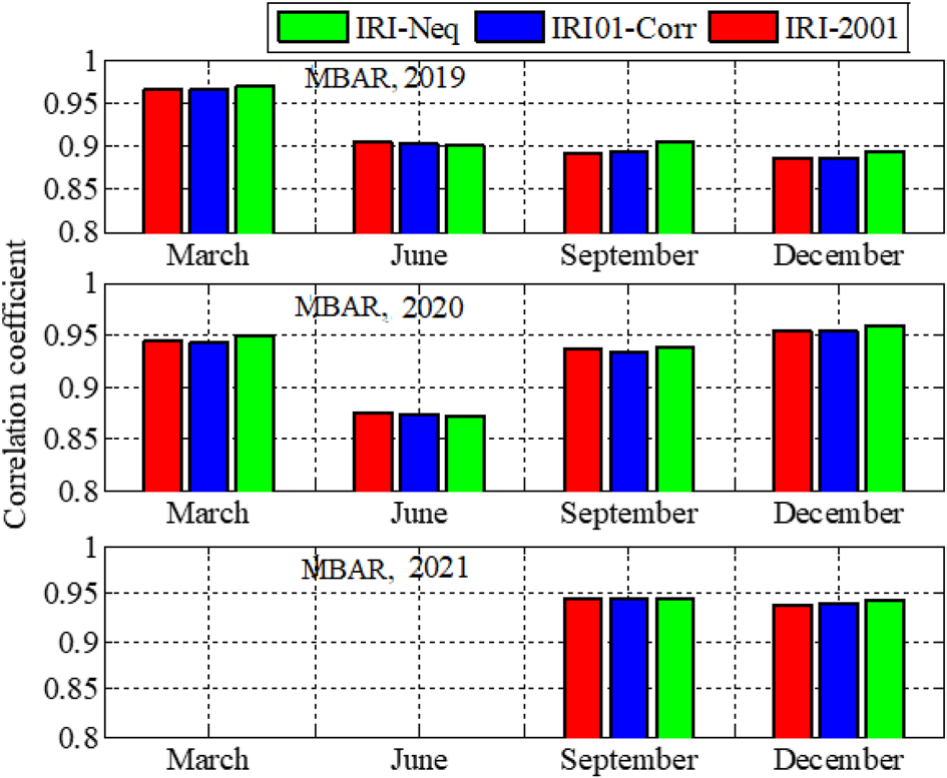
Correlation coefficients between the IRI-2016 TEC values and the GPS-TEC values using monthly means of the 5 quietest days for equinoxes and solstices months for MBAR.

**Figure 9:**
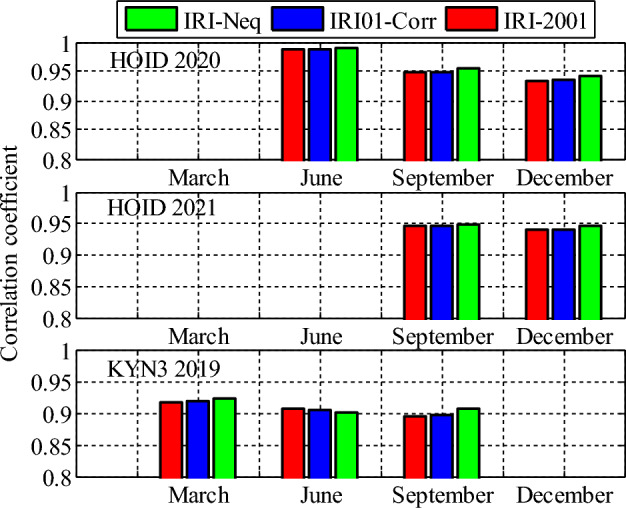
Correlation coefficients between the IRI-2016 TEC values and the GPS-TEC values using monthly means of the 5 quietest days for equinoxes and solstices months for HOID (2020 and 2021) and KYN3 (2019).

**Figure 10:**
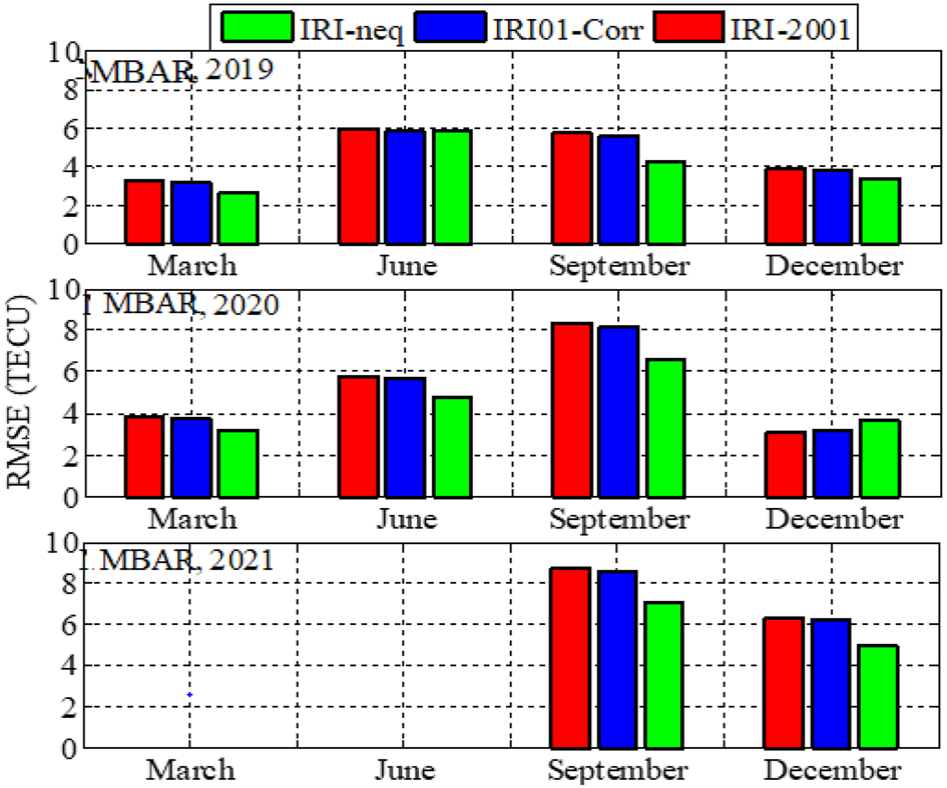
Root mean square errors of the IRI-2016 TEC values from the GPS-TEC values using monthly means of the 5 quietest days for equinoxes and solstices for MBAR.

**Figure 11:**
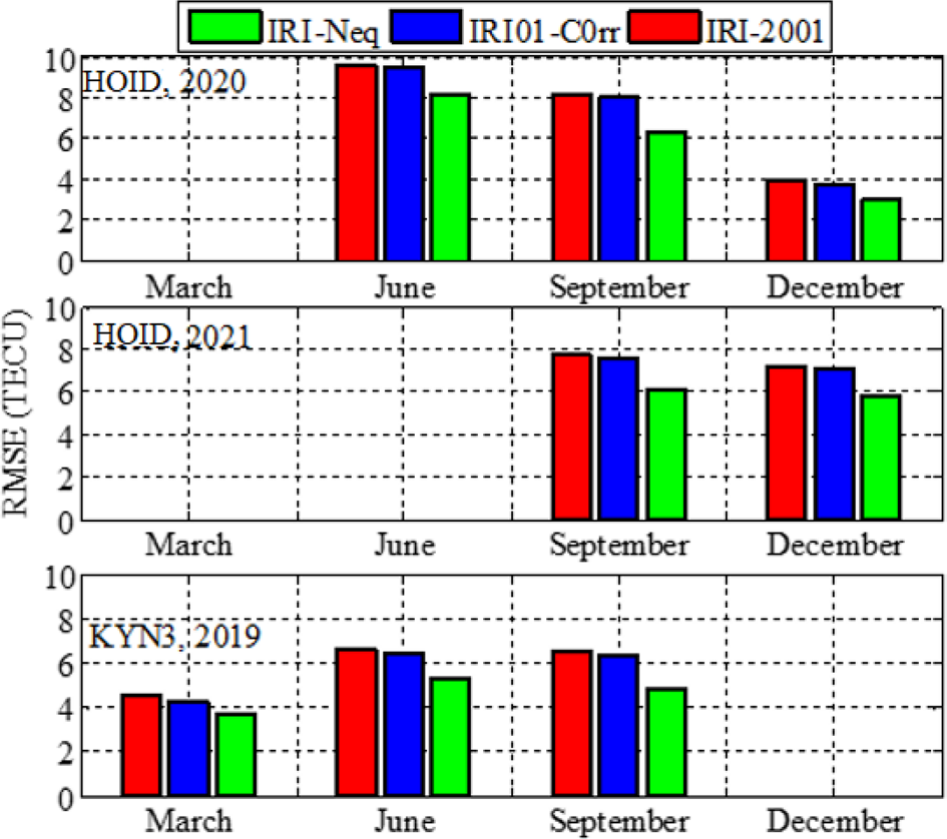
Root mean square errors of the IRI-2016 TEC values from the GPS-TEC values using monthly means of the five (5) quietest days for equinoxes and solstices for HOID (2020 and 2021) and KYN3 (2019).

**Figure 12:**
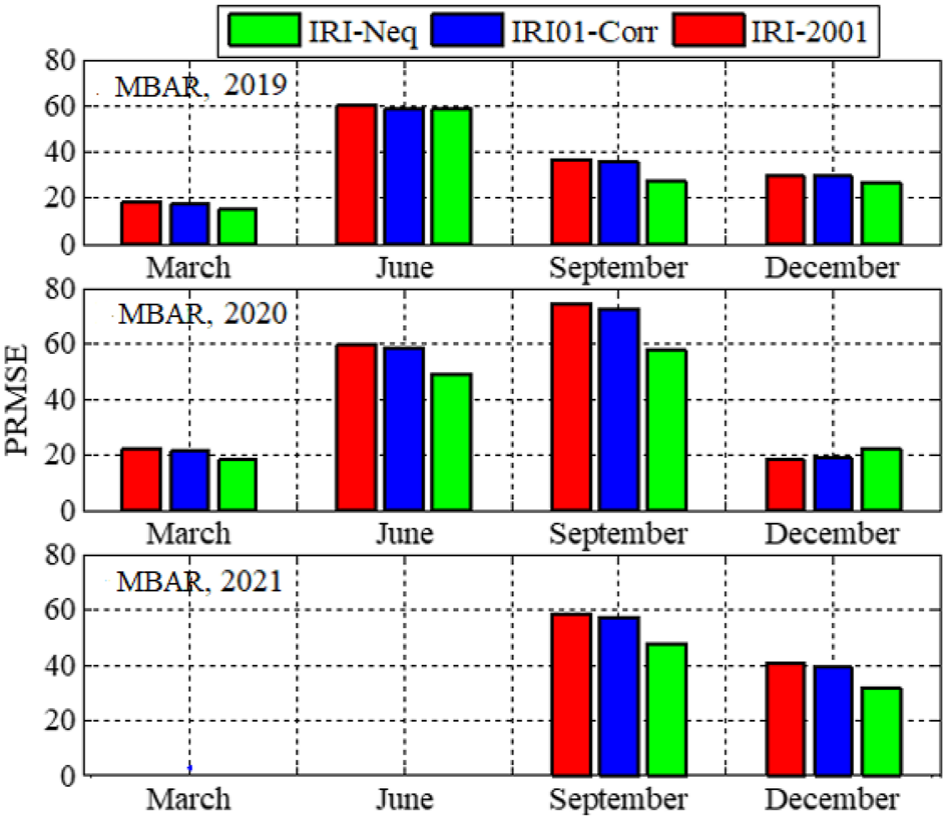
Percentage root mean square errors of the IRI-2016 TEC values from the GPS-TEC values using monthly means of the five (5) quietest days for equinoxes and solstices months for MBAR.

**Figure 13:**
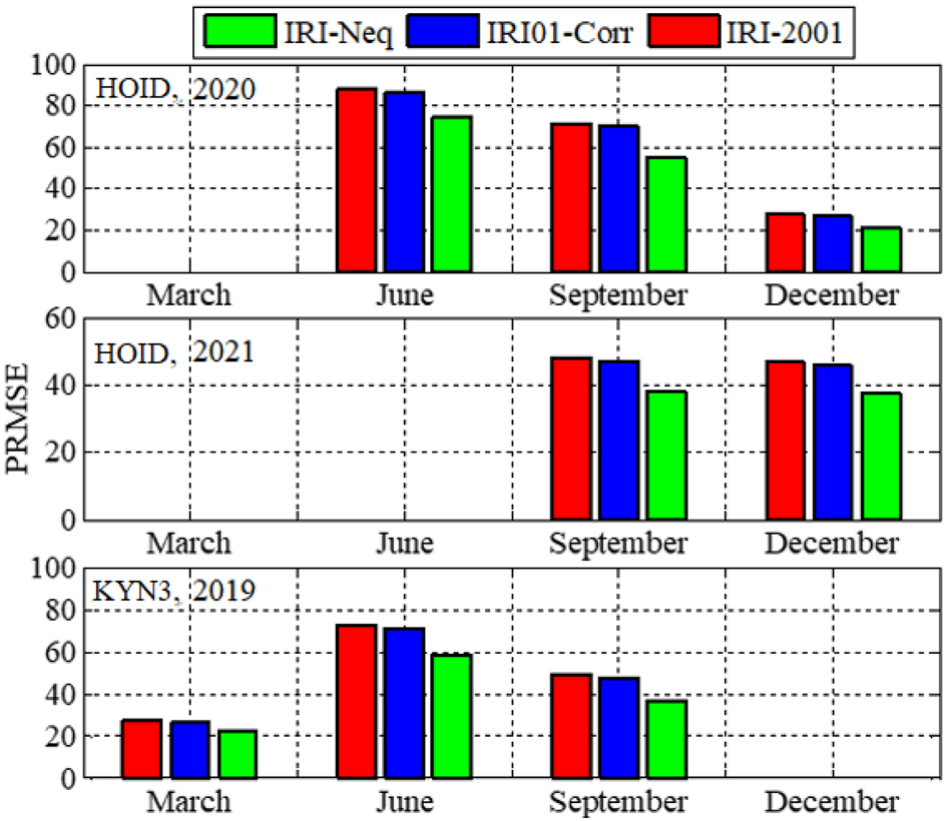
Percentage root mean square errors of the IRI-2016 TEC values from the GPS-TEC values using monthly means of the five (5) quietest days for equinoxes and solstices for HOID (2020 and 2021) and KYN3 (2019).

Analysis of GPS TEC and comparison with the TEC predicted by the IRI 2016 model for the equinoxes and solstices months from 2019 to 2021 was done at three stations located within the southern crest of the EIA at the Eastern Africa region. Figures 2, 3, 4, 5, 6, 7 compare the IRI TEC values for the three topside *Ne* options; IRI-Neq, IRI 2001, and IRI-01-Corr, with the diurnal values of GPS TEC for the given period. Using Eq. 4, correlation coefficients between the two sets of data were calculated (Figs. 8 and 9). The Root-Mean Square Errors (RMSE) of the IRI-TEC from the GPS-TEC (Fig. 10 and 11), and percentage RMSE of the IRI-TEC from the GPS-TEC (Figs. 12 and 13) were also calculated using (5) and (6).

The shape of the daily variations curves from GPS TEC and from all three options of the IRI-2016 model are similar, as can be seen in Figs. 2, 3, 4, 5, 6, 7. In all seasons, the TEC progressively rises from dawn, 3:00 to 4:00 UT (6:00 to 7:00 LT), peaking about noontime. At dusk, from 15:00 to 16:00 UT (18:00 to 19:00 LT), the TEC values start to fall, reaching their lowest points around midnight.

Figure 3 shows the comparison of GPS TEC and the TEC from IRI-2016 model at MBAR station using monthly means of the 5 quietest days of the equinoxes and solstices months for the year 2019. During March equinox for 2019, all the three topside *Ne* options of IRI-2016 model underestimated GPS-TEC from 0:00 UT to around 3:00 UT. From 3:00 UT to 7:00 UT, all the three topside *Ne* options overestimated the values of the GPS-TEC. After 7:00 UT, there were some overestimations and underestimations during certain periods of the day. For example, from 13:00 UT to 17:00 UT, there was an overestimation of GPS-TEC, and from 20:00 UT to 24:00 UT, there was an understimation. In addition, during March equinox, it was observed that IRI 2001 topside *Ne* option gave the lowest prediction values than other options.

For the June solstice of the year 2019, all the three topside *Ne* options of IRI-2016 model underestimated GPS-TEC from 0:00 UT to around 3:00 UT. From 3:00 UT to 16:00 UT, all the three topside *Ne* options overestimated the values of the GPS-TEC. However, from 16:00 UT to 24:00 UT, there was an overestimation of GPS-TEC by all IRI TEC values. Nevertheless, during June solstice, IRI 2001 topside *Ne* option was observed to give the lowest prediction values than other options. For the September equinox of the year 2019, all the three topside *Ne* options of IRI-2016 model had almost similar values with the GPS-TEC from 0:00 UT to around 3:00 UT. From 3:00 UT to around 9:00 UT, IRI-2016 model overestimated GPS-TEC, while from 10:00 UT to 12:00 UT, there was an underestimation of GPS-TEC by all the three topside *Ne* options of the IRI 2016 model.

The figure also shows that, IRI model overestimated the GPS-TEC from 13:00 UT to 19:00 UT, and from 20:00 UT to 24:00 UT, GPS TEC had close values to those estimated by IRI model. For the December solstice of the year 2019, all the three topside *Ne* options of IRI-2016 model had close values to those of the GPS-TEC from 0:00 UT to around 3:00 UT. From 3:00 UT to around 12:00 UT, IRI-2016 model showed an overestimation of GPS-TEC, while from 12:00 UT to 17:00 UT, there was an underestimation of GPS-TEC by all the three topside *Ne* options of the IRI 2016 model, and from 17:00 UT to 19:00 UT, IRI 2016 model overestimated the GPS-TEC. In addition, from 21:00 UT to 24:00 UT, IRI model slightly underestimated the GPS-TEC.

For the year 2020, as shown in Fig. 3, all the three options of the IRI 2016 model at MBAR have close values to GPS TEC values from 0:00 UT to around 3:00 UT for all seasons. From 3:00 UT to 13:00 UT, all the three topside *Ne* options overestimated the values of the GPS-TEC during March equinox. However, during this month, there was a close relationship between IRI TEC values and GPS TEC values between 14:00 UT and 20:00 UT, and overestimations from 20:00 UT to 24:00 UT. For June solstice, all the three topside *Ne* options overestimated the values of GPS TEC from 4:00 UT to 15:00 UT, and then there was underestimations from 15:00 UT to 24:00 UT. Similarly, in September equinox, all the *Ne* options overestimated the values of GPS TEC from about 3:00 UT to 19: 00 UT. Thereafter, from 19:00 UT to 23:00 UT, all the options showed slight underestimations. For December solstice, the performance of IRI model showed different behaviour as compared to other seasons. There were small overestimations and underestimations throughout. The significant underestimation was between around 9:00 UT to about 20:00 UT.

For the year 2021, only data for September equinox and December solstice were available at MBAR as shown in Fig. 4. For both seasons, IRI model showed almost similar trends throughout the day, although the curves showed different behaviours at different times. The notable trend was an overestimation which persisted almost from 2:00 UT to 24:00 UT, except at different periods such as at around 13:00 UT for December solstice where IRI TEC and GPS TEC had equal values.

Figure 5 shows a comparison of GPS TEC and IRI-2016 model TEC at Garba Tula (KYN3) for 2021. For this station, the data were available only during March, June and September of 2019. For March equinox, the IRI model underestimated GPS TEC from 00:00 UT to about 2:00 UT, thereafter, there was an underestimation to around 7:00 UT. From 7:00 UT to 12:00 UT, the GPS values had almost equal values to the IRI 2016 model predicted TEC. Then, the model showed an overestimation of GPS TEC up to around 19:00 UT, where again it underestimates the values of measured TEC up to 24:00 UT.

For June solstice and September equinox, the IRI model TEC and the GPS measured TEC had close values from 00:00 UT to 3:00 UT. However, for June solstice, there was an overestimation of GPS measured TEC by IRI model from 3:00 UT to about 15:30 UT. Thereafter, from 15:00 UT to 24:00 UT, the values of TEC predicted by IRI 2016 model using all topside *Ne* options, closely match the GPS measured TEC. On the other hand, during September equinox, the IRI predicted TEC had higher values as compared to measured TEC from around 3:00 UT to around 19:00 UT, and from about 19:00 UT to 24: 00 UT, the values of GPS TEC were higher than the predicted TEC.

Figures 6 and 7 show the comparison of GPS TEC and IRI-2016 model TEC at Hoima (HOID) for 2020 and 2021 respectively. Though, for 2020, the data were available only for June solstice, September equinox and December solstice, and for 2021, the data were available for only September equinox and December solstice. From Fig. 6, it is observed that, the IRI model exhibits the similar trends during June and September. From 00:00 UT to about 3:00 UT, predicted TEC by IRI model and GPS TEC have approximately equal values, and from about 3:00 UT to about 21:00 UT–23:00 UT, the IRI model overestimated the GPS values. From about 21:00 UT to 24:00 UT, the IRI model had close values to the GPS TEC. For December solstice, the difference between prediction during this month and the June solstice and September equinox prediction is seen only from around 10:30 UT to 16:00 UT where the values of GPS TEC and IRI 2016 TEC seem to have close values except at 15:000 UT where GPS TEC were higher than predicted TEC.

From Fig. 7, the values of predicted TEC by IRI 2016 using all topside *Ne* options and the GPS measured TEC had similar trends. The IRI model overestimated the values of GPS TEC throughout the day, except at around 10:00 UT–10:30 UT for September equinox and at around 13:00 UT for December solstice, where the values of IRI TEC and GPS TEC are close to one another.

In addition, in all the cases studied, it was observed that IRI 2001 topside *Ne* option gives the lowest prediction values than other options. Nevertheless, the IRI-Neq and IRI-01-corr topside *Ne* options observed to have approximately equal values during all seasons.

### Correlation coefficients, RMSE and PRMSE

Figure 8 shows the correlation coefficients between the IRI-2016 TEC values and the GPS-TEC values using the monthly means of the five quietest days during the equinoxes and solstices months for MBAR station which is at the southern crest of the EIA within the Eastern Africa region. The presented result is for the years 2019, 2020 and 2021. Correlation coefficients between the IRI-2016 TEC values and the GPS-TEC values during the equinoxes and solstices months for HOID (2020 and 2021) and KYN3 (2019) are shown in Fig. 9. The RMSE of the IRI-2016 TEC values from GPS derived TEC values for MBAR, HOID and KYN3 are presented in Figs. 10 and 11. Figures 12 and 13 presents information on the PRMSE of the IRI-2016 TEC values from GPS-TEC values for the same stations. However, there were no data at these stations for some months as shown in figures which might be due to infrastructure failures such as electrical power shutdowns or due to maintenance reasons during those particular periods.

From Figs. 8 and 9, it is observed that, GPS TEC values and IRI-TEC values for all the three topside *Ne* options show very good correlation. At most of the stations and during all seasons where data were available, correlation coefficient is above 0.9, which is quite strong. However, correlation coefficients at other stations are below 0.9, but above 0.8.5 which are also good. For example, during September equinox at MBAR for 2019, correlation coefficient for IRI01-Corr and IRI-2001 are below 0.9, and during December solstice, correlation coefficients for all topside *Ne* options are below 0.9 but above 0.85. For 2020, at MBAR station, it was observed that, correlation coefficients during June solstice for all the three topside *Ne* options were below 0.9, but above 0.85. In addition, for KYN3 2019, the correlation coefficient for IRI01-Corr and IRI-2001 during September equinox were observed to be slightly less than 0.9.

Figures 10 and 11 show the RMSE of IRI-TEC from GPS TEC at three stations, MBAR, HOID and KYN3 for the years 2019, 2020 and 2021. On the other hand, Figs. 12 and 13 show PRMSE for the respective stations and years. Nevertheless, there are missing data for some months as seen in the figures. The TEC using IRI-Neq had small RMSE than other options except during December, 2020 at MBAR where IRI-Neq RMSE were higher as compared to other options. At MBAR station, RMSE during September equinox of 2020 and 2021 were higher, above 8 TECU, reaching above 70% and 60% for IRI-2001 and IRI01-Corr *Ne* options respectively. The lowest errors were observed to occur during March equinox at all stations and for all months where the data were available. The PRMSE were observed to range from 18 to 25% at all stations and for all years where data were available. It should be noted that, some months had no data as it can be seen in presented figures. Generally, from all the stations and during all years, IRI-Neq was observed to have less RMSE, whereas IRI-2001 was observed to have maximum RMSE, having PRMSE of above 80% during June solstice of 2020 at HOID Station. Okoh et al.^10^ found that the IRI TEC values mostly related well with the GPS TEC values, with correlation coefficients nearly about 0.9, and root-mean square deviations almost about 20–50% for diurnal evaluations. This situation of IRI-Neq to have the smallest deviations of IRI-TEC values from GPS TEC values compared to IRI-2001 was also reported by Rathore et al.^24^ at Varanasi, India.

## Discussion

The TEC from the IRI-2016 model, which includes all the three topside *Ne* options (IRI-2001, IRI-Neq, and IRI-01-corr), has been compared with GPS-derived TEC from ground observations at three stations located within the southern crest of the EIA in the Eastern Africa region.

The results show that, from sunrise, 3:00 to 4:00 UT (6:00 to 7:00 LT), the TEC gradually increases, reaching its peak at noon. The TEC levels decrease around sunset, from 15:00 to 16:00 UT (18:00 to 19:00 LT), and they peak at midnight. Generally, all the three topside *Ne* options of the IRI-2016 model underestimated GPS-TEC during night hours, and during the day time there was an overestimation of GPS-TEC values by IRI-2016 model. However, there were varies overestimations and underestimations at different times at different stations. On the other hand, there was a different trend at MBAR and HOID during September and December, 2021, where IRI-2016 was observed to overestimate GPS-TEC throughout the day. The performance of the IRI model in the equatorial region was also reported by Mengistu et al.^2^, where they demonstrated that the apparent discrepancy between the GPS-TEC and IRI predicted TEC is caused by incorrectly calculated indices (namely, F10.7) that are utilized as the model's drivers at the regional level. Chartier et al.^25^ and Tariq et al.^19^ reported that, O/N2 ratio variation may potentially be the cause of the IRI TEC deviation from the GPS TEC. This variation arises from lower thermosphere plasma drift that moves upward. They further suggested that, the topside layer variabilities brought on by these processes might be mitigated by including thermospheric features in the IRI model. Okoh et al.^10^ reported that, relatively small amount of data from the region under consideration during model's development may have influenced the departures of IRI TEC from GPS TEC. This is because the accuracy of the IRI model in a particular location is dependent upon the availability of data for that region. In addition, according to Bilitza and Reinisch^26^, the lack of ionospheric data over the African equatorial region is a factor that possibly responsible for the smaller precisions of the IRI predictions over the region.

On the other hand, an underestimation of GPS TEC by IRI TEC during the night and overestimation during the day was also obtained by various scholars^2,27–29^, and associated this by the percentage of plasmaspheric contribution to GPS TEC which is much larger during the nighttime hours than the daytime hours. The contribution is high in the equatorial region because of the long distance the GPS ray travels through the plasmasphere^19^. Other researchers such as^30–32^ had an opinion that, the underestimating of the IRI TEC values in comparison to the GPS TEC may be caused by the enrichment of the plasmaspheric electron content over 2,000 km while the IRI model did not take the plasmaspheric component into account. According to Okoh et al.^10^, underestimations of GPS TEC by IRI TEC occurs because the upper integration limit of 1000 km selected for the IRI predictions may not always align precisely with the upper integration limit of the GPS calibration procedure. Adebiyi et al.^33^ attributed the TEC data-model offset to the height limitation of the IRI model, the inaccurate predictions of the electron density profiles, and the plasmaspheric contribution to TEC in the region. Tsidu and Zegeye^34^ reported that, the IRI model is robust in detecting observed TEC over EIA crest regions at the extreme ends despite the high RMSE. Consequently, the performance of the IRI-2016 model at the extremes of the observed TEC distribution highlights the necessity of more work to enhance the model in order to enable real-time operational forecasting.

From this study therefore, small accuracy of IRI TEC from GPS TEC might be possibly influenced by incorrectly calculated indices (namely, F10.7) that are utilized as the model's drivers at the regional level. Also, O/N2 ratio variation may have potentially influenced the IRI TEC deviation from the GPS TEC.

## Conclusions

I have examined the TEC variations from the IRI-2016 model at three stations positioned in the southern crest of the Eastern Africa region. This was done by utilizing all three topside Ne options (IRI-neq, IRI-2001, and IRI-01-corr). Using the monthly means of the five international quiet days during the equinoxes and solstices months of 2019–2021, the IRI TEC and the GPS observations were compared for the analysis. The correlation coefficients between the two sets of data, as well as the RMSE and the PRMSE of the IRI-TEC from the GPS-TEC were also calculated.

In general, the three topside *Ne* options of the IRI-2016 model underestimated GPS-TEC during the nighttime, whereas the model overestimated GPS-TEC values during the daytime. On the other hand, there were various overestimations and underestimations at various stations at various times. However, there was a different pattern in MBAR and HOID between September and December of 2021, where it was noted that IRI-2016 overestimated GPS-TEC all day long. It was also observed that, GPS TEC values and IRI-TEC values for all the three topside *Ne* options show very good correlation. At most of the stations and during all seasons where data were available, correlation coefficient was above 0.9, which is quite strong. The TEC using IRI-Neq had small RMSE than other options except during December, 2020 at MBAR where IRI-Neq RMSE were higher as compared to other options. Generally, from all the stations and during all years, IRI-Neq was observed to have less RMSE, reaching PRMSE of less than 20%, whereas IRI-2001 was observed to have maximum RMSE, having PRMSE of above 80% during June solstice of 2020 at HOID Station.

Thus, this work suggests that the low accuracy of IRI TEC from GPS TEC may be caused by the percentage of plasmaspheric contribution to GPS TEC which is much larger during the nighttime hours than the daytime hours. The contribution is high in the equatorial region because of the long distance the GPS ray travels through the plasmasphere.

## Data Availability

The datasets used during this study is available from the corresponding author upon request**.**
